# Assessing the relationship between community resilience and health outcomes: an observational local-authority level study in England

**DOI:** 10.1136/jech-2025-224513

**Published:** 2025-11-07

**Authors:** Christine Camacho, Peter Bower, Roger T Webb, Luke Munford

**Affiliations:** 1 Division of Population Health, The University of Manchester, Manchester, UK; 2 National Institute for Health and Care Research Applied Research Collaboration Greater Manchester, Manchester, UK; 3 Division of Psychology and Mental Health, The University of Manchester, Manchester, UK

**Keywords:** PUBLIC HEALTH, Health inequalities, HEALTH

## Abstract

**Background:**

Community resilience is a relevant concept in public health, but its empirical relationship with health outcomes remains underexplored. This study examines whether a Community Resilience Index (CRI) is associated with population health outcomes in England, and whether it offers explanatory added value beyond the Index of Multiple Deprivation (IMD).

**Methods:**

The CRI comprises 44 indicators reflecting community-level resilience to chronic stressors. Associations between CRI scores and five health outcomes, deaths of despair, cardiovascular disease (CVD) mortality, COVID-19 mortality, excess all-cause mortality during two waves of COVID-19 and self-rated health were assessed at local authority district level. IMD was adjusted to remove health-related indicators. Linear regression models assessed the explanatory power of the CRI and IMD, using likelihood ratio tests to compare model fit. Interaction and stratified analyses explored effect modification by IMD.

**Results:**

Higher CRI scores were associated with lower Deaths of Despair and CVD mortality, and higher self-rated health; these associations remained significant after adjusting for IMD. CRI was not significantly associated with COVID-19 outcomes. IMD remained the stronger predictor of health outcomes, but CRI significantly improved model fit. The interaction between CRI and IMD was significant for deaths of despair and self-rated health. Stratified analyses showed the CRI–deaths of despair association was strongest in more deprived areas.

**Conclusions:**

Community resilience is associated with health outcomes in England. While not a substitute for deprivation-based measures, resilience indices offer complementary insight into structural and social factors shaping health. Resilience-building efforts may be particularly impactful in areas of greatest disadvantage.

**WHAT IS ALREADY KNOWN ON THIS TOPIC**

- The association between community resilience and population health outcomes is poorly understood, with limited empirical evidence.

**WHAT THIS STUDY ADDS**

- This study shows that community resilience is associated with deaths of despair, cardiovascular disease mortality and self-rated health, adds explanatory value beyond deprivation and interacts with deprivation to influence health outcomes.

**HOW THIS STUDY MIGHT AFFECT RESEARCH, PRACTICE OR POLICY**

- Community resilience measures may help identify priority areas for place-based interventions to reduce health inequalities.

## Introduction

 Community resilience refers to the collective abilities and capacities of people and organisations within a place, to withstand, adapt to and recover from shocks.^1 2^ Although originally framed around disaster preparedness, its relevance to public health is increasingly recognised. Resilience is typically viewed as a dynamic process shaped by internal assets (eg, social capital, economic resources) and external supports (eg, governance, infrastructure).^3^ It is a multidimensional concept, encompassing social, economic, institutional, infrastructural and environmental domains.^4^ These domains are central to shaping population health outcomes, positioning community resilience as an important lens for place-based public health approaches.

The Disaster Resilience of Place (DROP) model conceptualises resilience as the interaction between inherent community characteristics and adaptive capacities in response to specific hazards.^5^ For public health, chronic stressors such as poverty, inequality and poor living conditions often dominate over acute events. Community resilience and public health share a common focus on the social determinants of health, including income, education, housing and social capital.^6 7^ Resilience-building can be seen as a preventative public health strategy, addressing spatial inequalities and supporting communities facing structural disadvantage.^8^

Empirical evidence linking community resilience to health outcomes remains limited, partly due to challenges in measurement.^9^ Composite indices have emerged as the predominant approach to measuring resilience at the community level. However, most existing indices, including the widely cited Baseline Resilience Indicators for Communities (BRIC),^10^ were developed in the context of natural hazards and are primarily used to assess preparedness and recovery in acute disaster settings. The Community Resilience Index (CRI) for England was developed to address this gap, adapting the BRIC framework for chronic stressors.^11^ Indicators were selected based on relevance to the English context, data availability at local authority level and exclusion of health outcomes to avoid circularity.^12^

While the CRI aligns conceptually with social determinants of health, its relationship to population health and established deprivation measures is not fully understood. The Index of Multiple Deprivation (IMD) remains the primary area-level measure of disadvantage in England and is strongly associated with poor health outcomes.^13 14^ However, deprivation measures offer limited insight into the assets that help communities thrive. Analysis of ‘left behind’ neighbourhoods suggests that places with weaker civil infrastructure may experience poorer health outcomes despite similar levels of deprivation.^15^ As public health increasingly adopts asset-based approaches, there is growing interest in whether community resilience might buffer the health impacts of deprivation.^16^

In this study, the concepts of resilience and deprivation are distinguished. The CRI is an asset-oriented measure that is underpinned by the DROP framework. The purpose of the CRI is to capture the antecedent place-based capacities that enable populations to cope with and adapt to chronic stressors. By contrast, the IMD is a deficit-oriented measure that summarises unmet need. The IMD is informed by the theory that people can be relatively deprived across multiple domains compared with social norms.^17^ While both measures intersect with the social determinants of health (eg, education, employment, housing),^18^ the CRI additionally incorporates civic and institutional capacities (eg, voluntary sector presence, local fiscal position), connectivity/digital access and inequality structure. We, therefore, perceived the CRI and IMD to be complementary and have explicitly tested whether the CRI adds information beyond the IMD. Strengthening community assets could help narrow area-level health inequalities; accordingly, we tested whether CRI–health associations vary across the deprivation spectrum.

This study examines whether community resilience, as measured by the CRI, is associated with population health outcomes in England, by addressing three questions:

1. Is community resilience associated with deaths of despair (DoD), cardiovascular disease (CVD) mortality, COVID-19 mortality, excess all-cause and self-rated health? (RQ1)
2. Does including the CRI to regression models alongside the IMD improve explanatory power? (RQ2)
3. Does the association between community resilience and health outcomes vary across levels of deprivation? (RQ3)

## Methods

This ecological analysis was conducted for 307 local authority districts (LADs) in England (2022 boundaries),^19^ excluding the City of London and Isles of Scilly due to small population sizes. The study used publicly available, anonymised area-level data and was exempt from ethical review.

### Health data sources

Five health outcomes were selected to reflect different dimensions of population health and their theorised links to community resilience. DoD were included as they are hypothesised to be linked to the breakdown of community structures following economic shocks, leading to long-term decline and cumulative disadvantage.^20^ DoD were defined as the sum of alcohol-specific, drug-related and suicide deaths using mutually exclusive International Statistical Classification of Diseases (ICD-10) groupings aligned with Office for National Statistics (ONS) definitions: alcohol-specific E24.4, F10, G31.2, G62.1, G72.1, I42.6, K29.2, K70, K85.2, K86.0, Q86.0, R78.0, X45, X65, Y15; drug-related F11–F16, F18–F19, X40–X44, X60–X64, X85, Y10–Y14; suicide (including undetermined intent) X66–X84, Y16–Y34, with alcohol-/drug-specific codes excluded from the suicide category to ensure no overlap. DoD were measured using directly age-standardised mortality rates per 100 000 population, calculated for each LAD over the period 2019–2021.^21^

COVID-19 was included as a recent example of an external shock, given that community resilience frameworks propose that resilient communities are better able to withstand and recover from such shocks.^22^ COVID-19 outcomes included age-standardised mortality rates attributed to COVID-19 (March 2020–April 2021, using ICD-10 codes U07.1, U07.2, U10.9),^23^ and excess all-cause mortality during the first two waves of the pandemic (March–June 2020 and September 2020–March 2021, compared with 2015–2019 averages).^24^ Excess deaths provide a broader measure of pandemic impact, capturing undiagnosed COVID-19 cases and deaths linked to healthcare disruption.^25^

CVD mortality was included to represent a chronic, non-communicable condition that is well known to be shaped by the social determinants of health.^14^ While not a community-level shock like pandemics or economic crises, resilience factors (eg, social cohesion, infrastructure) may influence disease management and health behaviours.^26^ Including CVD enabled comparison with COVID-19 mortality to explore whether resilience–health associations differ between communicable and non-communicable conditions. Age-standardised CVD mortality rates per 100 000 were obtained for 2019–2021; for 17 LADs affected by boundary changes, 2018–2020 estimates were used for consistency.^27 28^

Self-reported health was included as a broader measure of population well-being. Beyond disaster scenarios, the endpoint for resilient communities is debated; authors have proposed ‘thriving communities’ and better general health as the aspirational outcome of resilience-building.^22 29^ Consistent with that perspective, we treat improved population health as a plausible downstream expression of higher community resilience. Self-reported health was measured using data from the 2021 Census.^30^ Age-standardised proportions were used, and the percentage of the population reporting ‘good’ and ‘very good’ health was retained for analysis.

### Predictors

The main predictor was the CRI, comprising 44 indicators across 5 domains: Access and Infrastructure (CRI-1), Economic Wellbeing and Opportunity (CRI-2), Social Capital and Connectivity (CRI-3), Diversity and Inclusion (CRI-4) and Equity and Stability (CRI-5). Internal consistency was high overall (Cronbach’s α=0.93), with domain-level α of 0.92 (CRI-1), 0.92 (CRI-2), 0.79 (CRI-3); α=0.60 (CRI-4); 0.54 (CRI-5).

The CRI is conceptually grounded in the DROP framework and was developed using the Organisation for Economic Co-operation and Development (OECD) 10-step framework for composite index construction,^31^ adapted to the and chronic stressors (rather than acute hazards). Indicators were selected based on (1) applicability to the English context, (2) relevance to longer-term adversity, rather than specifically to acute hazards and (3) availability at LAD level. Population health outcomes were excluded to avoid circularity. Indicators were harmonised in direction (higher=greater resilience), transformed to percentile ranks and combined into subdomain scores using principal components. Subdomains were then aggregated using eigenvalue-based weights. Higher CRI scores indicate greater resilience. Full development details are provided in our prior paper^11^; the complete indicator list and definitions are in online supplemental table S1.

For comparison, the English IMD, a composite index composed of seven domains: income, employment, education, health, crime, barriers to housing and services, and living environment, was included.^32^ To avoid conceptual overlap with health outcomes, an adjusted IMD was calculated, setting the health domain weight to zero for analyses, while retaining the published weights for the remaining six domains.^33^ The official documentation for the IMD does not report an internal consistency statistic. Cronbach’s alpha was approximated by treating the six retained IMD domain scores as the items, yielding α=0.75.

As context, the two indices overlap at the level of broad themes (eg, education, employment, housing/access), but they are operationalised with different indicator sets and measurement choices. The CRI additionally incorporates constructs not covered by IMD (civic/voluntary sector and community assets, local government finance, inequality structure and digital/transport connectivity), whereas IMD includes a dedicated crime domain. A list of CRI and IMD domains and indicators is provided in online supplemental table S1.

### Statistical analysis

All analyses were conducted in Stata V.17 (StataCorp). Spatial patterns were described using choropleth maps based on SD bands relative to the national mean.^34^

For RQ1, health outcomes were summarised by CRI quintile using ANOVA, and Pearson correlations were computed for CRI, IMD and individual CRI domains. Correlation results were visualised in a heatmap.

For RQ2, linear regression models estimated associations between CRI and health outcomes, comparing models with CRI alone, IMD alone and both predictors combined. Likelihood ratio (LR) tests assessed whether adding CRI improved model fit. To explore any differences in explanatory power, subindex level analyses were included. For each outcome, IMD-only baseline was compared with models adding either the overall CRI or one CRI subindex (S1–S5) using nested regression models and LR tests. Results are reported as the change in R-squared (ΔR² = R²_full − R²_base) and LR p values.

For RQ3, interaction terms (CRI×IMD) were included alongside CRI and IMD main effects to test for effect modification. Predictors were mean-centred to reduce multicollinearity.^35^ LR tests compared models with and without interaction terms. Where significant, stratified models were estimated by IMD quintile to explore how CRI-health associations varied across the deprivation gradient. Variance inflation factors (VIFs) were calculated for regression models including both CRI and IMD, and for models including the CRI×IMD interaction term, to assess potential multicollinearity.

## Results

As hypothesised, higher CRI scores were generally associated with lower rates of adverse outcomes (DoD, CVD mortality) and higher self-rated health, while higher IMD scores (greater deprivation) showed the opposite pattern. Descriptive maps of outcome rates are provided in online supplemental figure S1.

### Association between CRI and health outcomes (RQ1)

Health outcomes across CRI quintiles showed clear gradients for DoD, CVD mortality and self-rated health, with better outcomes for more resilient areas (online supplemental table S2). Mean DoD rates fell from 40.6 to 26.1 per 100 000 between the least and most resilient quintiles. Similar trends were observed for CVD mortality (248.9 vs 199.9 per 100,000) and self-rated health (80.0% vs 84.3%). All differences were statistically significant (p<0.001). No clear gradient was observed for COVID-19 mortality (p=0.087) or excess deaths (p=0.76).

**Figure 1:**
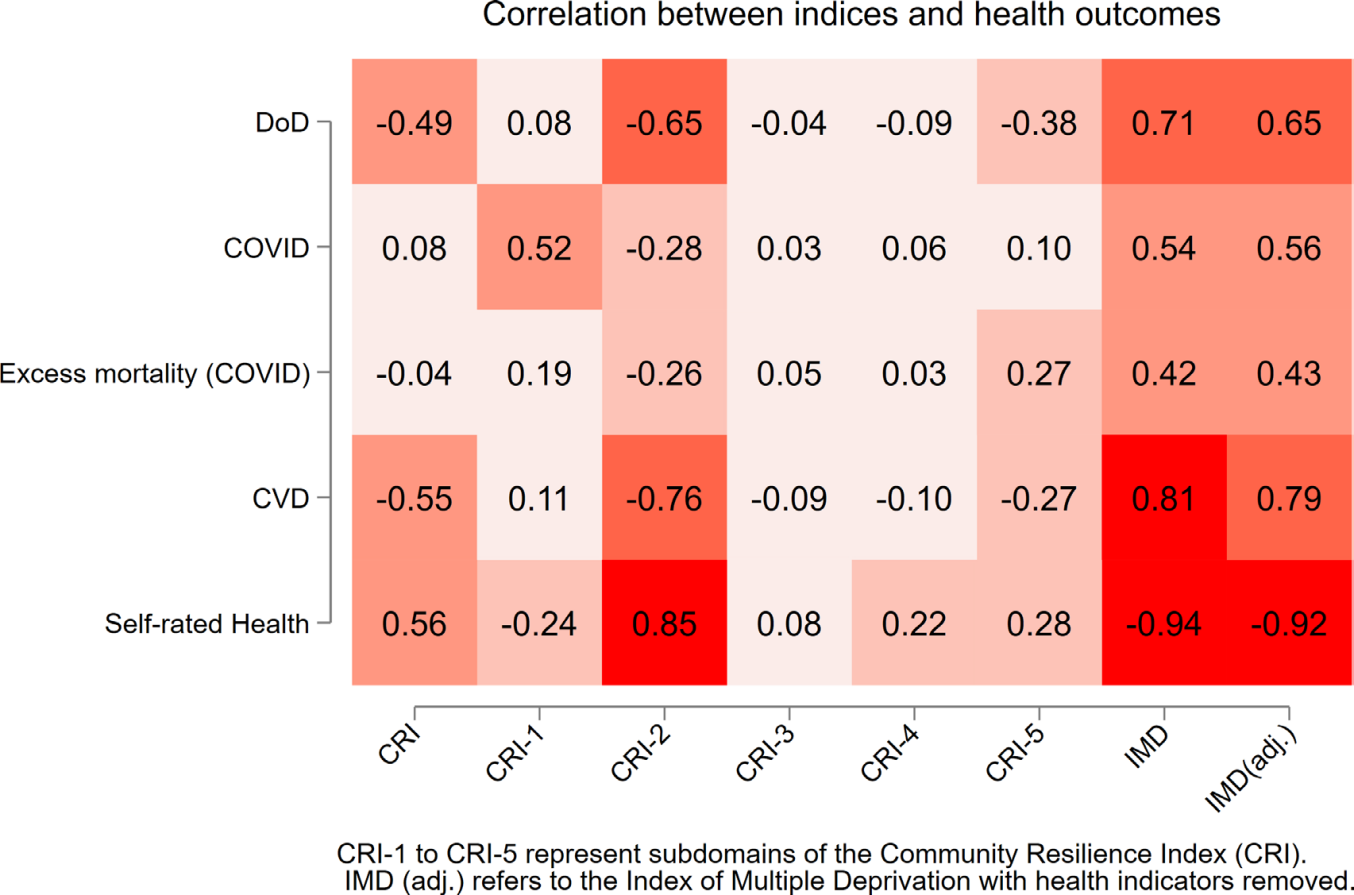
Heatmap showing correlations between indices and health outcomes. CVD, cardiovascular disease; DoD, deaths of despair; IMD, Index of Multiple Deprivation.

Correlation analysis confirmed these patterns. Adjusted IMD showed the strongest correlations, particularly with self-rated health (r=–0.92) and CVD mortality (r=0.79). CRI-2 (Economic Wellbeing and Opportunity) showed strong correlations with self-rated health (r=0.85), CVD mortality (r = –0.76) and DoD (r=–0.65), while CRI-1 (Access and Infrastructure) was positively correlated with COVID-19 mortality (r=0.52), counter to expectations (figure 1).

In unadjusted linear regression models, higher CRI was associated with lower DoD (β=–0.49, 95% CI −0.59 to −0.39, p<0.001), lower CVD mortality (β=–1.60, 95% CI −1.87 to −1.32, p<0.001) and better self-rated health (β=0.16, 95% CI 0.13 to 0.18, p<0.001), but associations with COVID-19 or excess mortality were not evident (table 1). IMD was significantly associated with all outcomes and consistently explained a greater proportion of variance than CRI.

**Table 1:** Regression coefficients and R² values for models predicting health outcomes using either the Community Resilience Index (CRI) or adjusted Index of Multiple Deprivation (IMD) as sole predictors

Outcome	Predictor(s)	β coefficient (95% CI)	R^2^
Deaths of despair	CRI	−0.49*** (−0.59 to 0.39)	0.24
	IMD	1.12*** (0.98 to1.27)	0.43
COVID-19	CRI	0.48 (−0.21 to1.16)	0.01
	IMD	5.69*** (4.73 to 6.65)	0.31
Excess mortality	CRI	−1.08 (-3.86 to 1.69)	0.00
	IMD	18.02*** (13.80 to 22.24)	0.19
CVD	CRI	−1.60*** (−1.87 to 1.32)	0.30
	IMD	3.91*** (3.57 to 4.24)	0.63
Self-rated good health	CRI	0.16*** (0.13 to 0.18)	0.32
	IMD	−0.43*** (−0.45 to to 0.41)	0.85

* p<0.05, **p<0.01, ***p<0.001.

CVD, cardiovascular disease.

### Explanatory value of CRI beyond IMD (RQ2)

For all outcomes, combined models including both CRI and IMD improved fit compared with IMD-only models (table 2). In the combined models, both predictors remained independently associated with DoD, CVD mortality and self-rated health. The largest increase in explanatory power was observed for COVID-19 mortality (R² increasing from 0.31 to 0.50), a pattern consistent with statistical suppression—a predictor (CRI) with weak bivariate association to the outcome improves fit when entered with a correlated predictor (IMD) because it accounts for shared variance with that predictor.^36^ The CRI component not shared with IMD (ie, CRI residual conditional on IMD) is positively associated with COVID-19 mortality. Multicollinearity was low (mean VIF=1.38).

**Table 2:** Regression coefficients and adjusted R² values for CRI+IMD models; LR tests comparing IMD only to CRI+IMD models

Outcome	Predictors	β coefficient (95% CI)	R^2^	LR test IMD vs CRI+IMD
Deaths of despair	CRI	−0.20*** (−0.30 to 0.10)	0.46	χ^2^ =15.3, p=0.0001
	IMD	0.95*** (0.78 to 1.11)		
COVID-19	CRI	3.10*** (2.53 to 3.67)	0.5	χ^2^ =98.5, p<0.0001
	IMD	8.43*** (7.47 to 9.39)		
Excess mortality	CRI	6.25*** (3.39 to 9.11)	0.23	χ^2^ =18.1, p<0.0001
	IMD	23.54*** (18.72 to 28.36)		
CVD	CRI	−0.53*** (−0.75 to 0.30)	0.65	χ^2^ =20.4, p<0.0001
	IMD	3.44*** (3.06 to 3.82)		
Self-rated good health	CRI	0.03*** (0.02 to 0.04)	0.86	χ^2^ =18.8, p<0.0001
	IMD	−0.40*** (−0.42 to to 0.38)		

* p<0.05, **p<0.01, ***p<0.001.

CRI, Community Resilience Index; CVD, cardiovascular disease; IMD, Index of Multiple Deprivation; LR, Likelihood ratio.

The subindex analysis showed that the Equity and Stability (S5) subindex contributed across every outcome (largest increments for DoD and excess mortality), and Economic Wellbeing and Opportunity subindex (S2) also contributed consistently; other subindices showed outcome-specific effects (eg, Access and Infrastructure (S1) for COVID-19). Detailed ΔR² and LR statistics are shown in online supplemental table S3.

### Interaction between CRI and IMD (RQ3)

**Figure 2:**
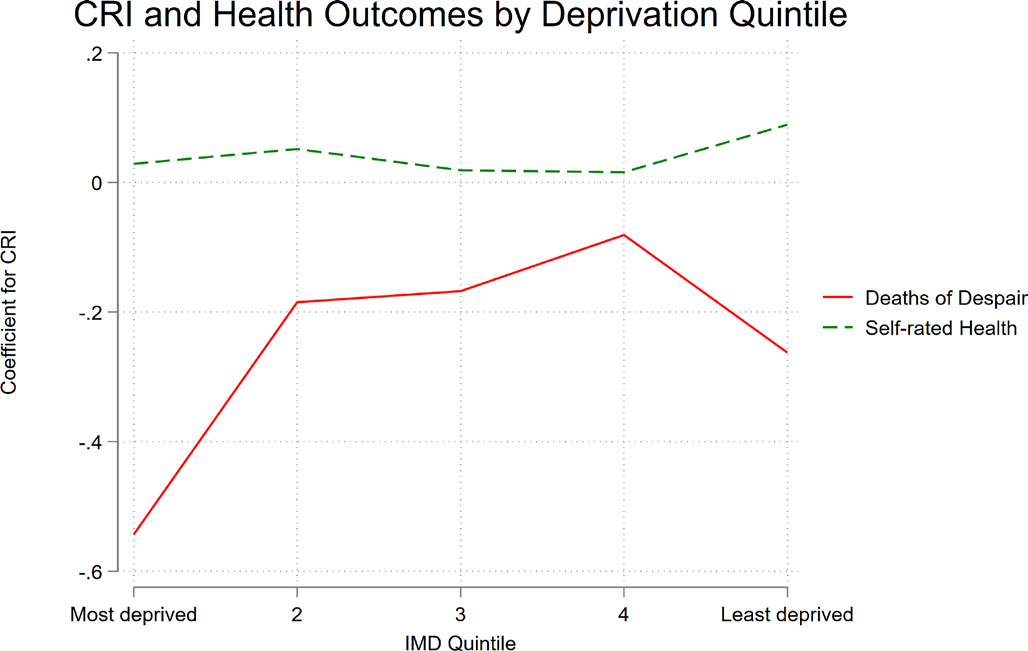
Association between the CRI and health outcomes across IMD quintiles. CRI, Community Resilience Index; IMD, Index of Multiple Deprivation.

Models including CRI×IMD interaction terms showed significant effect modification for DoD (p=0.02) and self-rated health (p=0.01), but not for COVID-19, excess mortality or CVD (table 3). VIFs remained low (mean VIF=1.27), indicating minimal multicollinearity. Stratified analyses (figure 2) showed that for DoD, the association with CRI was strongest in the most deprived quintile (β=–0.54), weaker and non-significant in quintiles 2–4, and smaller but significant in the least deprived quintile (β=–0.26). For self-rated health, CRI was positively associated in the least deprived quintile (β=0.09, 95%) and weaker (quintile 2, β=0.05) and non-significant in other quintiles.

**Table 3:** Regression coefficients and adjusted R² values for CRIxIMD models; likelihood ratio (LR) tests comparing CRI+IMD to CRIxIMD models

	β coefficient (95% CI)				
	CRI	IMD	CRIxIMD	R^2^	LR test CRI+IMD vs CRIxIMD
Deaths of despair	−0.20***	0.95***	−0.02*	0.47	χ^2^=5.86, p=0.02
	(−0.30 to 0.10)	(0.78 to 1.11)	(−0.03 to 0.00)		
COVID-19	3.11***	8.43***	0.04	0.5	χ^2^=1.17, p=0.28
	(2.54 to 3.68)	(7.47 to 9.38)	(−0.04 to 0.12)		
Excess mortality	6.24***	23.54***	−0.03	0.23	χ^2^=0.02, p=0.90
	(3.37 to 9.11)	(18.72 to 28.37)	(−0.43 to 0.38)		
CVD	−0.53***	3.44***	−0.03	0.66	χ^2^=2.76, p=0.10
	(−0.76 to 0.31)	(3.06 to 3.82)	(−0.06 to 0.01)		
Self-rated good health	0.03***	−0.40***	−0.002*	0.87	χ^2^=6.23, p=0.01
	(0.02 to 0.04)	(−0.42 to 0.38)	(−0.004 to 0.001)		

* p<0.05, **p<0.01, ***p<0.001.

CRI, Community Resilience Index; CVD, cardiovascular disease; IMD, Index of Multiple Deprivation.

## Discussion

The CRI was significantly associated with DoD, CVD mortality and self-rated health. These associations remained after adjusting for deprivation, indicating that the CRI captures community-level factors relevant to population health not reflected in deprivation measures. By contrast, no significant associations were found between CRI and COVID-19 mortality or excess all-cause mortality during the first two pandemic waves. These findings suggest that the CRI aligns more closely with chronic, socially patterned outcomes than acute impacts. While IMD consistently explained a greater proportion of variance, CRI improved model fit for all outcomes. Significant CRI×IMD interactions were observed for DoD and self-rated health, with stratified analyses showing the CRI–health association varied across the deprivation gradient.

The overall CRI score is not associated with COVID-19 mortality and excess mortality. Subindex analyses showed heterogeneity: Access and Infrastructure (S1) was positively associated with COVID-19 mortality conditional on IMD, consistent with connectivity and mobility increasing exposure risk. Two subindices contributed additional explanatory power across all outcomes: Equity and Stability (S5) (conceptually distinct from IMD) and Economic Wellbeing and Opportunity (thematically overlapping with IMD but measured differently). These findings reflect the CRI’s multidimensional nature and point towards different domains being more relevant for specific outcomes, indicating targeted application.

### Interpretation in the context of existing evidence

This study provides initial evidence that community resilience, as measured through a composite index, is associated with several important health outcomes in England. The CRI appears to capture structural and social conditions relevant to long-term health inequalities, reinforcing established evidence on the social determinants of health.^13 14^ These findings provide empirical support for the role of community resilience as a place-based determinant of population health.

Similar associations have been reported in studies of social capital. For example, Poortinga found that bonding, bridging and linking social capital were positively associated with self-rated health but did not significantly buffer the effects of area-level deprivation.^37^ While conceptually related, our findings diverge in showing significant interactions between community resilience and deprivation for certain outcomes, suggesting that a multidimensional index may be better suited to detecting these effects.

The enduring impact of deprivation on health outcomes was consistent with previous research, but our results also highlight variation among equally deprived areas. This echoes findings from studies such as Walsh *et al*’s comparison of Glasgow, Manchester and Liverpool, which concluded that deprivation alone cannot fully explain spatial patterns in premature mortality.^38^ Our findings align with this, suggesting that community-level factors captured by the CRI may account for some of this unexplained variation.

The lack of association between CRI and COVID-19 mortality was initially surprising but aligns with emerging evidence that some features typically considered resilience assets may increase risk in the context of an acute infectious disease. An international study of COVID-19 mortality across 84 countries found that while income inequality was consistently associated with higher mortality, certain forms of social capital, such as social trust and group membership, were associated with greater mortality.^39^

### Public health and policy implications

Deprivation indices such as the IMD remain the dominant tools for identifying and targeting health inequalities. However, they offer a limited view of what enables communities to sustain well-being. The CRI provides a complementary lens, highlighting structural and social conditions that may not be captured by deprivation alone. The significant interaction between CRI and deprivation in relation to DoD suggests that community resilience may play a particularly protective role in the areas facing the greatest structural disadvantage. For those commissioning and delivering place-based interventions, this highlights the value of strengthening community resilience alongside addressing material deprivation.

Recent policy agendas echo this approach. The All-Party Parliamentary Group on Left Behind Neighbourhoods called for greater devolution, investing in social infrastructure and consolidation of fragmented place-based funding.^40^ Similarly, the UK Government Resilience Framework emphasises prevention and the importance of building baseline resilience across systems to manage future risks.^41^ While the framework focuses primarily on acute disruptions, its principles are equally relevant to long-term public health challenges, where persistent inequalities are not only markers of social injustice but also indicators of communal fragility.

In this context, measures such as the CRI could support system leaders in identifying priority areas for investment. Directors of Public Health, with their statutory duty to improve population health and reduce inequalities, are well positioned to lead resilience-building efforts. Targeting investment towards community-level assets and capacities offers a pragmatic route to delivering on national commitments, including the UK Labour Party’s pledge to tackle the social determinants of health and halve the life expectancy gap between richest and poorest areas.^42^ While the CRI was developed for the English context, the broader concept of community resilience as a modifier of deprivation-related health outcomes may be applicable to other countries, though context-specific adaptation of measures would be required.

### Strengths and limitations

To our knowledge, this is the first study to examine associations between a composite CRI and population health in England. While similar indices exist, we are not aware of comparable analyses that have empirically tested associations between community resilience and health outcomes at a population level. The study adds value by testing whether the CRI offers explanatory insight beyond the IMD. The CRI was independently associated with several key outcomes, and its inclusion improved model fit for all outcomes. By using multiple outcomes and testing for interaction effects, the study went beyond asking whether resilience is associated with health to explore under what conditions these associations are strongest.

There are several limitations to this study. First, the study is ecological at local authority level. Associations may not reflect individual-level relationships (ecological fallacy) or generalise to other spatial contexts. Use of secondary administrative data introduces potential measurement error; aggregation to LADs can dilute within-area variation and mask heterogeneity. Second, the analysis was cross-sectional, limiting causality inference. Mechanisms such as reverse causality (poor health eroding community resilience) and selective migration (out-migration of healthier residents and/or in-migration of those in poorer health) could bias area-level associations. Third, the health outcomes span the COVID-19 period, which may have introduced atypical patterns. Fourth, we did not examine outcomes such as child health, premature mortality or service access, which may also relate to resilience. Fifth, while CRI and IMD were treated as distinct, conceptual overlap is possible. Finally, excess mortality was treated as a continuous variable, though derived from count data. Poisson models were not used due to negative excess values.

### Unanswered questions and future research

This study highlights several important areas for further research. The analysis was cross-sectional and conducted at local authority level, limiting causal inference and potentially obscuring variation at smaller spatial scales. Future work could redevelop the CRI at finer geographic resolution, for example, middle layer super output areas (~7000 residents) or lower layer super output areas (~1500), to assess whether patterns hold at more granular levels. Longitudinal analyses would be valuable for exploring how resilience develops over time, assessing the impact of migration flows and testing whether changes in community resilience predict shifts in health outcomes, particularly in the context of chronic stressors or policy interventions. There is also potential to explore additional health outcomes, including premature mortality, healthy life expectancy, infant mortality and service access measures, to build a fuller picture of how community resilience relates to population health.

While the CRI added explanatory value beyond deprivation, the underlying mechanisms remain unclear. The subindex analysis offers some initial insights but does not distinguish distinct capacities from richer measurement of shared determinants. Future work could apply variance partitioning to decompose unique vs shared variance for the IMD and each CRI subindex. Social epidemiologists have theorised that relative deprivation can damage the social fabric of communities and have negative impacts on population health.^43 44^ Mediation analyses could be used to test whether the CRI (or specific CRI domains) mediate IMD–health associations. Finally, replication of the CRI framework in other parts of the UK or internationally could help assess its generalisability and inform the development of place-based approaches in diverse settings.

## Conclusions

This study offers initial evidence that resilience-based measures can complement deprivation indices in understanding population health. The CRI highlights structural and social conditions not captured by deprivation alone, and its association with key health outcomes suggests that it may help identify where the capacity to sustain well-being is weakest. In particular, the strong association observed between resilience and DoD in the most deprived areas points to the potential value of resilience-building as a targeted, preventative approach. By recognising longstanding health inequalities as markers of systemic fragility, resilience-based measures could support more nuanced, place-based strategies aimed at reducing risk and promoting health equity.

## Supplementary material

10.1136/jech-2025-224513 online supplemental figure 1

10.1136/jech-2025-224513 online supplemental table 1

10.1136/jech-2025-224513 online supplemental table 2

10.1136/jech-2025-224513 online supplemental table 3

## Data Availability

Data are available in a public, open access repository.
