# Supplementary material for: Assessing the relationship between community resilience and health outcomes: an observational local-authority level study in England
Source: J Epidemiol Community Health. 2025 Nov 7;80(2):e224513. doi: 10.1136/jech-2025-224513 (PMC12911620; doi:10.1136/jech-2025-224513)
Supplement: online supplemental figure 1 [file jech-80-2-s001.docx]

**Supplementary Figure S1 – Spatial distribution of health outcomes by local authority in England, grouped by standard deviation from the mean**

**
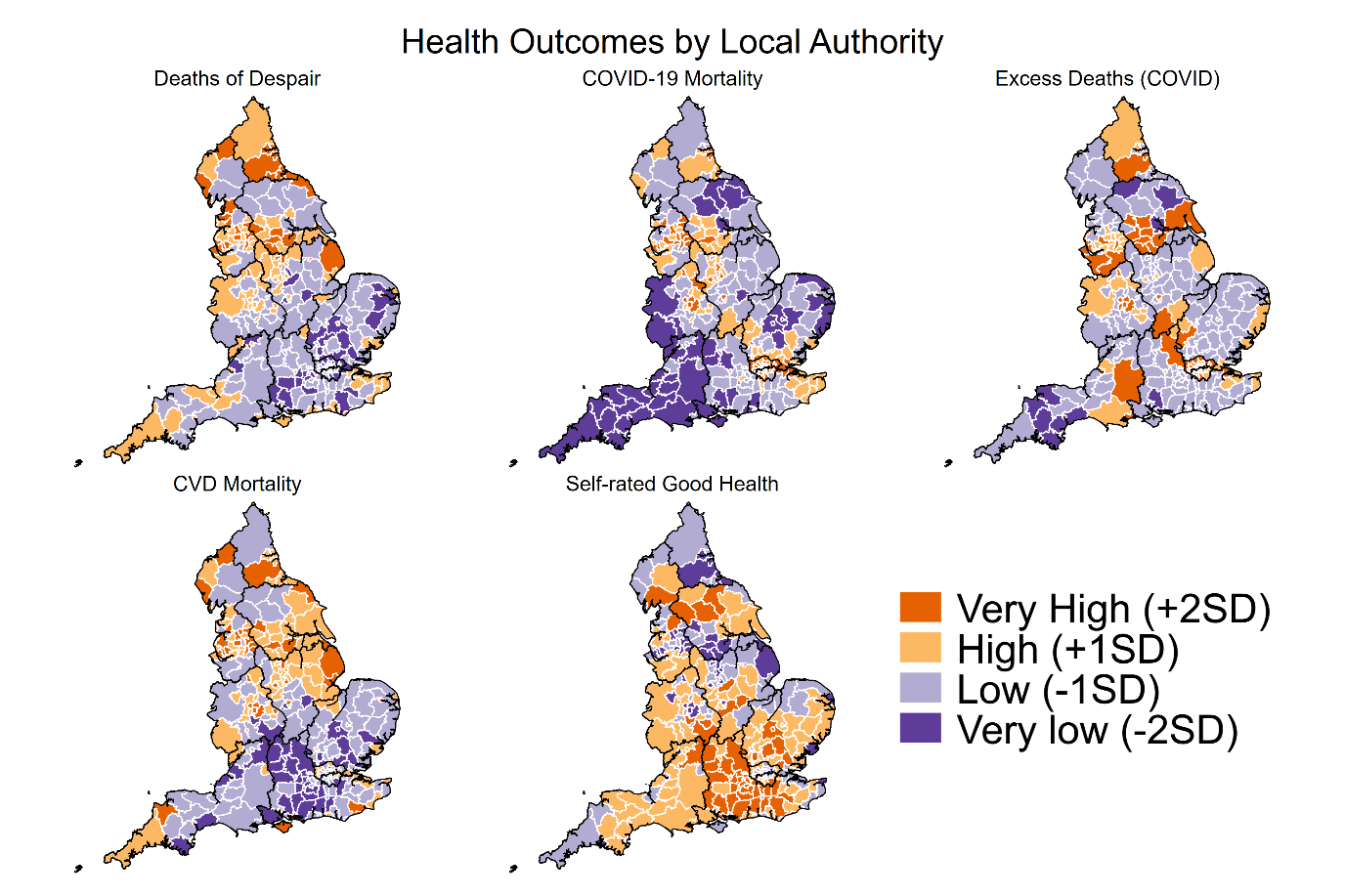
**
