# Supplementary material for: Assessing the relationship between community resilience and health outcomes: an observational local-authority level study in England
Source: J Epidemiol Community Health. 2025 Nov 7;80(2):e224513. doi: 10.1136/jech-2025-224513 (PMC12911620; doi:10.1136/jech-2025-224513)
Supplement: online supplemental table 1 [file jech-80-2-s002.docx]

**Supplementary Table S1** **– List of domains and indicator for Community Resilience Index and Index of Multiple Deprivation (excluding the health domain)**

| **Community Resilience Index** | |
| --- | --- |
| **Domain (Weighting)** | **Indicators** |
| Access and Infrastructure (12.5%) | Car availability Population density Household overcrowding Age dependency ratio Travel time to key services Public health grant Distance to sport and leisure facilities Private outdoor space Employment |
| Economic Well-being and Opportunity (8.0%) | Housing affordability Adult skills Child poverty Fuel poverty Gross disposable household income Electoral turnout Digital propensity Pupil absence Gross value added Inward migration Economic inactivity |
| Social Capital and Connectivity (3.5%) | SME loans Broadband access Lottery funding Charities Air pollution Noise complaints Public green space Assets of community value High street vibrancy |
| Diversity and Inclusion (2.4%) | Religious affiliation English language proficiency Living alone Municipal spending on culture Employment sector diversity Households in temporary accommodation Life satisfaction |
| Equity and Stability (1.6%) | Non-ringfenced reserves Core spending power IMD gap School quality Gender pay gap Gini index Population churn Food insecurity |

| **Index of Multiple Deprivation** | |
| --- | --- |
| Income Deprivation (22.5%) | Adults and children in Income Support families Adults and children in income-based Jobseeker’s Allowance families Adults and children in income-based Employment and Support Allowance families Adults and children in Pension Credit (Guarantee) families Adults and children in Working Tax Credit and Child Tax Credit families, below 60% median income not already counted Asylum seekers in England in receipt of subsistence support, accommodation support, or both Adults and children in Universal Credit families where no adult is in ‘Working - no requirements’ conditionality regime |
| Employment Deprivation (22.5%) | Claimants of Jobseeker’s Allowance, aged 18-59/64 Claimants of Employment and Support Allowance, aged 18-59/64 Claimants of Incapacity Benefit, aged 18-59/64 Claimants of Severe Disablement Allowance, aged 18-59/64 Claimants of Carer’s Allowance, aged 18-59/64 Claimants of Universal Credit in the ‘Searching for work’ and ‘No work requirements’ conditionality groups |
| Education, Skills & Training Deprivation (13.5%) | Key Stage 2 attainment: scaled scores Key Stage 4 attainment: average capped points score Secondary school absence Staying on in education post 16 Entry to higher education Adults with no or low qualifications, aged 25-59/64 Adults who cannot speak English or cannot speak English well, aged 25-59/64 |
| Crime (9.3%) | Recorded crime rates for:  • Violence  • Burglary  • Theft  • Criminal damage |
| Barriers to Housing & Services (9.3%) | Road distance to a:  • Post office  • Primary school  • General store or supermarket  • GP surgery Household overcrowding Homelessness Housing affordability |
| Living Environment Deprivation (9.3%) | Houses without central heating Housing in poor condition Air quality Road traffic accidents |
