# Supplementary material for: Assessing the relationship between community resilience and health outcomes: an observational local-authority level study in England
Source: J Epidemiol Community Health. 2025 Nov 7;80(2):e224513. doi: 10.1136/jech-2025-224513 (PMC12911620; doi:10.1136/jech-2025-224513)
Supplement: online supplemental table 2 [file jech-80-2-s003.docx]

**Supplementary Table S2 - Health outcomes by Community Resilience Index (CRI) quintiles**

| CRI quintile | Number of LADS | ASMR DoD per 100,000 (2019-2021) | ASMR COVID per 100,000 (2020-2021) | Excess deaths during COVID waves 1-2 | ASMR CVD per 100,000 (2019-2021) | Age standardised percentage of population reporting good or very good health (2021) |
| --- | --- | --- | --- | --- | --- | --- |
| Least resilient | N=62 | 40.55 (11.25) | 161.14 (70.71) | 360.16 (382.08) | 248.95 (29.72) | 79.99 (2.12) |
| 2 | N=61 | 37.53 (10.80) | 192.97 (70.79) | 376.16 (270.33) | 234.74 (28.66) | 80.32 (2.70) |
| 3 | N=62 | 35.68 (9.99) | 185.97 (60.99) | 331.81 (237.41) | 227.25 (27.12) | 81.73 (2.57) |
| 4 | N=61 | 27.61 (8.50) | 179.93 (67.39) | 323.51 (202.72) | 212.17 (24.64) | 83.41 (2.36) |
| Most resilient | N=61 | 26.07 (6.16) | 183.31 (55.14) | 325.20 (205.94) | 199.90 (23.01) | 84.32 (2.57) |
| p-value (ANOVA) |  | <0.001 | 0.087 | 0.76 | <0.001 | <0.001 |
| Data are presented as mean (SD)  LADs = local authority districts; ASMR = age-standardised mortality rate per 100,000 population. COVID waves 1–2 refer to March–June 2020 and September 2020–March 2021. | | | | | | |
