# Supplementary material for: Assessing the relationship between community resilience and health outcomes: an observational local-authority level study in England
Source: J Epidemiol Community Health. 2025 Nov 7;80(2):e224513. doi: 10.1136/jech-2025-224513 (PMC12911620; doi:10.1136/jech-2025-224513)
Supplement: online supplemental table 3 [file jech-80-2-s004.docx]

**Supplementary Table S3 - Added explanatory power of CRI and its subindices conditional on IMD. Cells show the change in R² vs the IMD-only base model (ΔR² = R²_full − R²_base) for each outcome; LR test p-values for the nested comparison are in brackets**

|  | CRI | S1 | S2 | S3 | S4 | S5 |
| --- | --- | --- | --- | --- | --- | --- |
| DoD | 0.03 (<0.001)*** | 0.00 (0.798) | 0.05 (<0.001)*** | 0.01 (0.06) | 0.00 (0.237) | 0.07 (<0.001)*** |
| COVID | 0.19 (<0.001)*** | 0.20 (<0.001)*** | 0.06 (<0.001)*** | 0.00 (0.912) | 0.03 (<0.001)*** | 0.04 (<0.001)*** |
| Excess | 0.04 (<0.001)*** | 0.01 (0.015)* | 0.02 (0.008)** | 0.00 (0.621) | 0.01 (0.0156)* | 0.12 (<0.001)*** |
| CVD | 0.02 (<0.001)*** | 0.00 (0.880) | 0.05 (<0.001)*** | 0.02 (<0.001)*** | 0.01 (0.041)* | 0.02 (<0.001)*** |
| Self-rated health | 0.01 (<0.001)*** | 0.02 (<0.001)*** | 0.04 (<0.001)*** | 0.02 (<0.001)*** | 0.01 (0.283) | 0.02 (<0.001)*** |
| * p<0.05, ** p<0.01, *** p<0.001  S1 Access & Infrastructure; S2 Economic Wellbeing & Opportunity; S3 Social Capital & Connectivity; S4 Diversity & Inclusion; S5 Equity & Stability | | | | | | |
